# Refractory Orthostatic Hypotension After Cervical Laminoplasty: A Case Report

**DOI:** 10.7759/cureus.97412

**Published:** 2025-11-21

**Authors:** Ryo Takahashi, Koji Hayashi, Kosuke Misaki, Katsunori Mizuno

**Affiliations:** 1 Department of Rehabilitation Medicine, Fukui General Hospital, Fukui, JPN; 2 Department of Orthopedics, Fukui General Hospital, Fukui, JPN

**Keywords:** central cord syndrome, cervical spine injury, postural orthostatic hypotension, spinal cord injury, spine rehabilitation

## Abstract

We report a case of postoperative orthostatic hypotension (OH) after cervical laminoplasty for central cord syndrome (CCS). A 76-year-old man with a history of glaucoma sustained a fall and developed incomplete tetraparesis. Magnetic resonance imaging revealed severe cervical stenosis (C2-4 and C4-6) with intramedullary T2 hyperintensity, leading to a diagnosis of cervical compressive myelopathy. After initial conservative management and rehabilitation, where blood pressure remained stable (mean systolic blood pressure 127 mmHg, diastolic blood pressure 64 mmHg), he underwent C3-6 laminoplasty on day 41 from onset. From postoperative day 2, he experienced recurrent dizziness and transient loss of consciousness associated with significant systolic blood pressure drops (≥20 mmHg) during postural changes, confirming a diagnosis of OH. Despite comprehensive nonpharmacological interventions, including compression stockings, gradual orthostatic training with a tilt table, and recumbent bicycle exercise, his OH persisted, characterized by consistently low blood pressure during rehabilitation. Although no syncopal episodes occurred, the OH was considered refractory and lasted until discharge approximately seven months later. This case highlights that postoperative OH can be a significant and refractory complication in elderly patients with cervical compressive myelopathy after cervical laminoplasty, even with standard nonpharmacological rehabilitation. Increased awareness of this complication is crucial, and further research is needed to elucidate its underlying mechanisms and develop more effective management strategies.

## Introduction

Orthostatic hypotension (OH) is defined as an excessive fall in blood pressure upon changing from the supine to the upright position, internationally characterized as a decrease in systolic blood pressure (SBP) of ≥20 mmHg or diastolic blood pressure of ≥10 mmHg within 3 minutes of standing [1]. Typical symptoms include dizziness, unsteadiness, blurred vision, and syncope, which increase the risk of falls and trauma [1]. The underlying mechanisms involve impaired autonomic reflexes, reduced circulating blood volume, the effects of medication, and age-related decline in cardiovascular regulation [2]. OH is not merely a transient discomfort but has been associated with increased cardiovascular events and mortality, making it a clinically important manifestation of autonomic dysfunction [3]. 

Central cord syndrome (CCS), first described by Schneider in 1954, is a subtype of traumatic incomplete cervical spinal cord injury (SCI) [4]. It occurs predominantly in the elderly and is characterized by disproportionately greater motor impairment in the upper extremities than in the lower extremities [5]. Among non-fracture cervical spinal cord injuries, CCS is one of the most frequent and clinically significant patterns [6]. 

Cervical laminoplasty is a posterior decompression procedure widely performed for multilevel cervical compression, such as cervical spondylotic myelopathy and ossification of the posterior longitudinal ligament (OPLL). Developed in Japan in the 1970s, it allows expansion of the spinal canal while preserving posterior elements, in contrast to conventional laminectomy [7]. Techniques include the open-door and double-door methods, both aiming at sustained decompression of the spinal cord [8]. Laminoplasty has gained international acceptance as an effective procedure for multilevel disease, with long-term neurological improvement reported [9]. 

We report a case of a 76-year-old man with CCS who developed refractory OH following cervical laminoplasty. Recent systematic reviews highlight that the overall literature on OH management in the general SCI population is sparse and of poor quality [10]. Consequently, reports specifically detailing nonpharmacological interventions for OH after spinal surgery are particularly limited. While nonpharmacological measures, such as compression stockings and orthostatic training, are widely recommended as first-line treatments, their efficacy is highly questionable, especially in vulnerable populations like the elderly. Indeed, a comprehensive review focusing on older adults (≥65 years) concluded that there is no high-quality evidence (GRADE) supporting any nonpharmacological therapy, with only weak recommendations for compression therapy made based on very low-quality evidence [10]. This case underscores the importance of recognizing and managing postoperative OH in patients undergoing laminoplasty and highlights the need to deepen our understanding of its complex pathophysiology, given the current lack of established treatment strategies. 

## Case presentation

**Figure 1:**
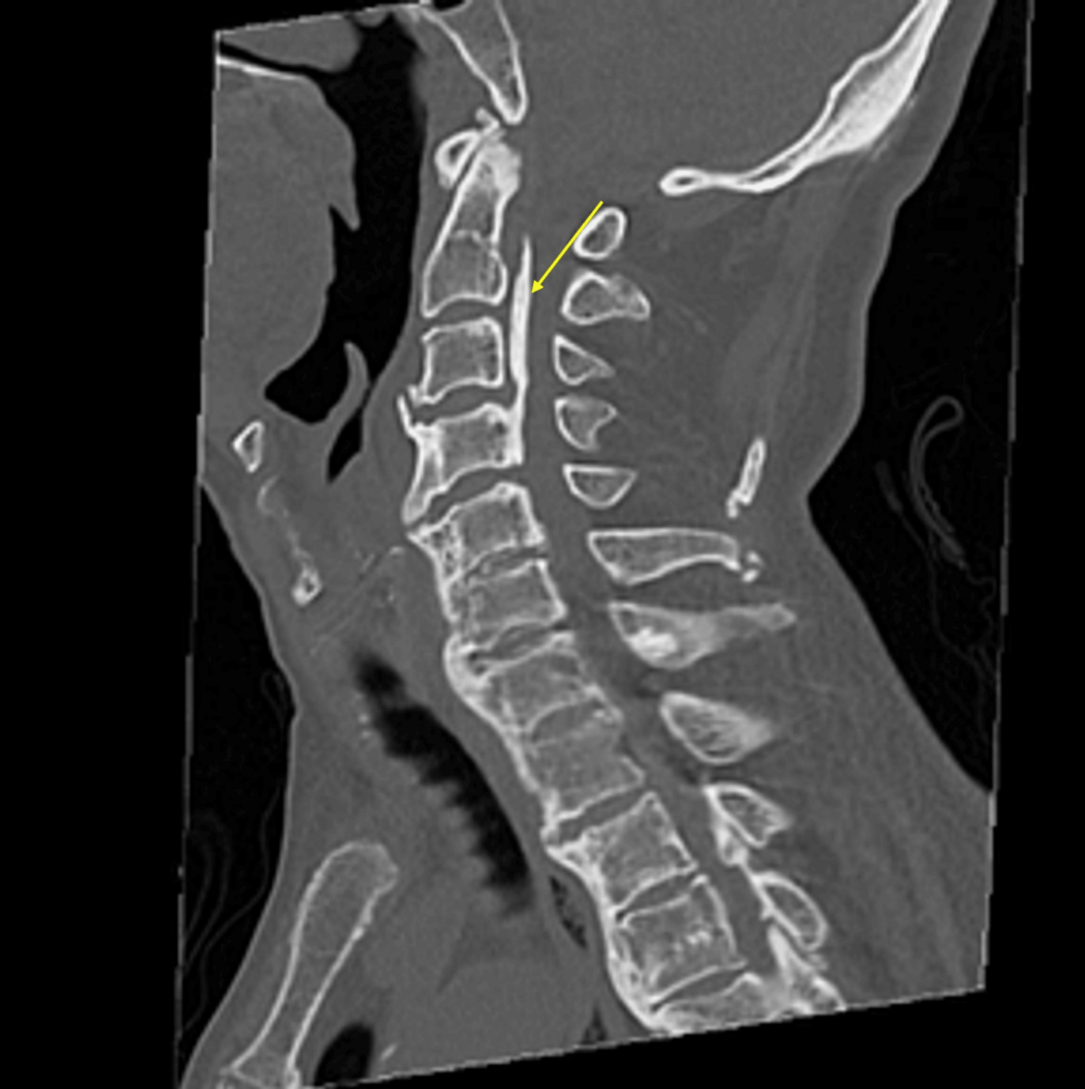
Cervical CT findings. Cervical CT shows calcification of the posterior longitudinal ligament (arrow). CT, computed tomography.

**Figure 2:**
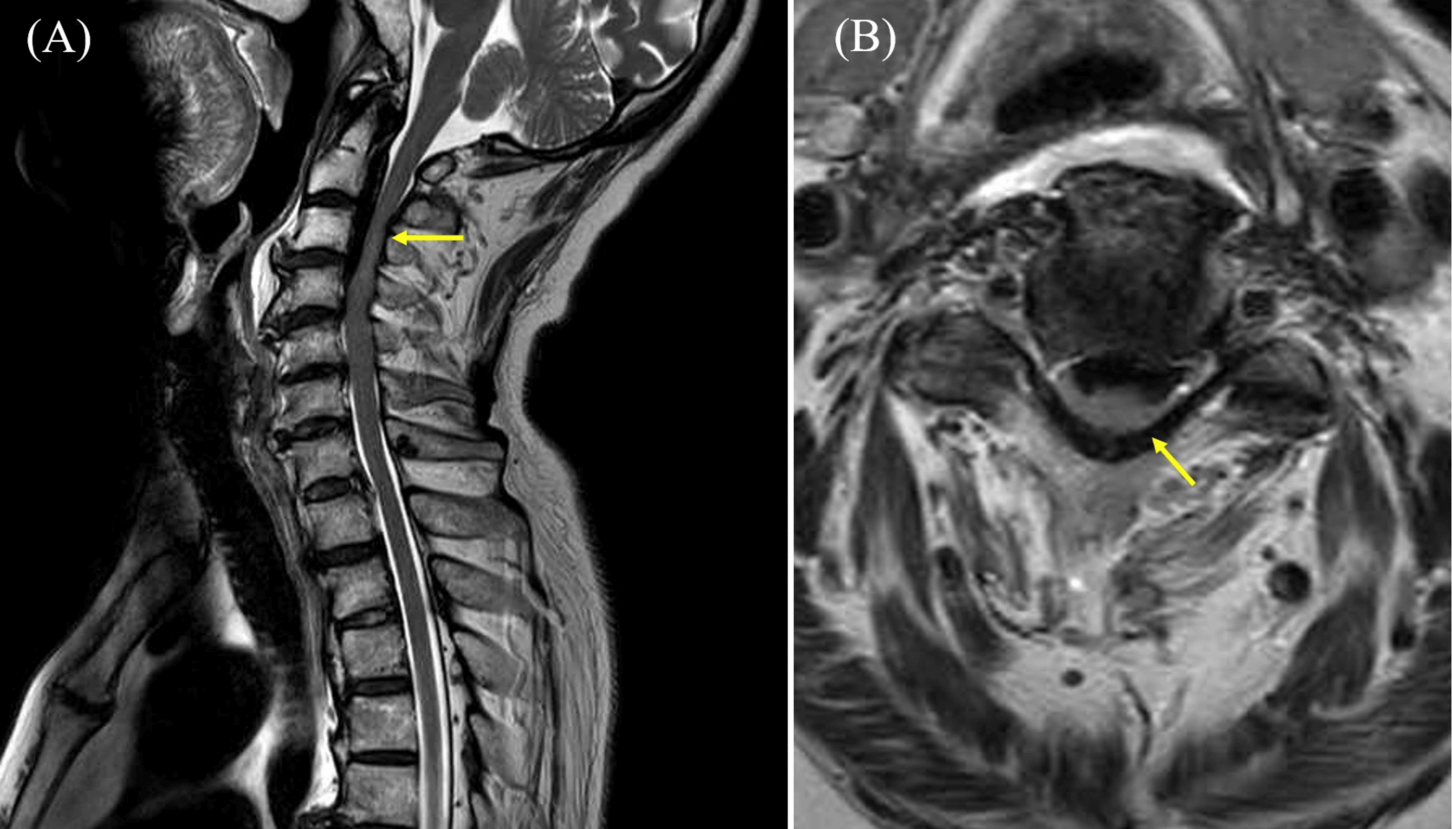
Cervical MRI findings on T2-weighted images. (A) Sagittal MRI of the cervical spine showing severe stenosis at C2-4, moderate stenosis at C4-6, and intramedullary T2 hyperintensity at the same levels (arrow). (B) Axial MRI at the C2 level demonstrating severe stenosis and hyperintensity of the cervical spinal cord (arrow). MRI, magnetic resonance imaging.

A 76-year-old man with a history of glaucoma sustained a fall and was initially evaluated at a previous hospital. Neurological examination revealed incomplete tetraparesis, with the Medical Research Council (MRC) grade 1-2 in the right upper limb, grade 2-4 in the left upper limb, and grade 3 in both lower limbs. Computed tomography (CT) demonstrated calcification in the posterior longitudinal ligament (Figure 1), and magnetic resonance imaging (MRI) showed severe stenosis at C2-4, moderate stenosis at C4-6, and intramedullary T2 hyperintensity at the same levels (Figure 2). Based on these findings, he was diagnosed with CCS. He was transferred to our hospital on day 16 from onset, and rehabilitation therapy was initiated the following day. During rehabilitation, his blood pressure remained stable, with a mean systolic pressure of 127 mmHg (range, 97-156 mmHg) and a mean diastolic pressure of 64 mmHg (range, 57-71 mmHg). These values are summarized in Table 1. Although surgery for CCS was recommended at the time of admission to our hospital, the family initially opted for conservative management, and rehabilitation was continued. However, neurological improvement was limited, and C3-6 laminoplasty was performed on day 41 from onset. 

**Table 1:** Blood pressure during rehabilitation before and after surgery BP, blood pressure.

Phase/Condition	Mean Systolic BP (mmHg)	Range (mmHg)	Mean Diastolic BP (mmHg)	Range (mmHg)	Notes
Preoperative rehabilitation	127	97-156	64	57-71	Hemodynamically stable
Postoperative rehabilitation	78	48-108	52	32-71	

From postoperative day 2 (hospital day 43), he experienced repeated episodes of dizziness and transient loss of consciousness associated with an SBP drop of ≥20 mmHg during sitting and postural changes (Table 1). Both active standing and head-up tilt testing [3] reproduced these findings, and OH was diagnosed according to international criteria [1]. No antihypertensive or autonomically active medications were administered perioperatively. On postoperative day 3 (hospital day 44), wheelchair training was initiated, and compression stockings were prescribed. Rehabilitation interventions included gradual orthostatic training using a tilt table from postoperative day 21 (hospital day 62) and recumbent bicycle exercise from postoperative day 31 (hospital day 72). A concise timeline of symptom onset and rehabilitation interventions is presented in Table 2. Rehabilitation sessions were conducted five days per week, with two 60‑minute sessions per day. The program included wheelchair training, tilt‑table orthostatic training, and recumbent bicycle exercise, adjusted according to the patient’s tolerance. Despite these interventions, his blood pressure during rehabilitation remained low, with a mean systolic pressure of 78 mmHg (range, 48-108 mmHg) and a mean diastolic pressure of 52 mmHg (range, 32-71 mmHg). Although he suffered from OH, no syncopal episodes occurred during hospitalization. His OH persisted for approximately seven months after surgery despite multiple non-pharmacological interventions and was regarded as refractory. Although his motor function improved clinically to the extent that he was able to use a wheelchair, the ASIA motor and Functional Independence Measure (FIM) scores did not show measurable improvement. At discharge, the FIM motor score was 46/91 and the cognitive score was 35/35, indicating preserved cognitive independence but severely limited motor function. This discrepancy indicates that persistent postoperative OH significantly limited functional recovery despite rehabilitation efforts. The patient's entire course timeline is summarized in Table 2.

**Table 2:** Timeline of symptom onset OH, orthostatic hypotension; CCS, central cord syndrome.

Days From Onset	Hospital Day	Event/Intervention	Notes
Day 0	–	Fall injury	Incomplete tetraparesis developed
Day 16	Hospital Day 1	Transferred to our hospital; rehabilitation started the next day	Preoperative blood pressure stable
Day 41	Hospital Day 26	C3-6 laminoplasty performed	Surgery for CCS
Day 43 (Post-op Day 2)	Hospital Day 28	Onset of OH (dizziness, transient loss of consciousness)	BP drop ≥20 mmHg with sitting/position change
Day 44 (Post-op Day 3)	Hospital Day 29	Wheelchair training initiated; compression stockings prescribed	OH persisted
Day 62 (Post-op Day 21)	Hospital Day 47	Tilt-table orthostatic training started	OH persisted
Day 72 (Post-op Day 31)	Hospital Day 57	Recumbent bicycle exercise started	OH persisted
~7 months later	–	Discharge	

## Discussion

This report highlights the clinical importance of postoperative OH following cervical laminoplasty for CCS. The severity of his cervical SCI was classified as grade C according to the American Spinal Injury Association (ASIA) Impairment Scale (AIS). Following rehabilitation therapy after surgery, his paralysis improved, and he was able to use a wheelchair. However, since OH did not improve despite various nonpharmacological interventions, including compression stockings, gradual orthostatic training, and recumbent bicycle exercise, it was considered refractory OH. First, OH developed postoperatively, despite stable preoperative blood pressure. Second, the patient was elderly, and age-related autonomic decline may have contributed to the persistence of OH. Third, the OH followed a prolonged and refractory course, lasting approximately seven months despite multiple nonpharmacological interventions. These features highlight the clinical importance of postoperative OH in elderly patients with cervical SCI and underscore the need for tailored management strategies.

OH is a common problem in patients with SCI during the acute and early post-acute rehabilitation phases, particularly in those with tetraplegia [2,11]. Postural changes during physiotherapy and mobilization have been shown to induce clinically significant hypotensive episodes in 73.6% of patients with acute SCI, with symptoms occurring in 58.9% of these episodes [11]. Another study focusing on the first month following acute SCI found that 60% of individuals exhibited OH, predominantly in patients with cervical and upper thoracic motor complete SCI [12].

Additionally, cervical spine surgery itself is a risk factor for OH. A retrospective study reported that 22 out of 190 patients (11.6%) developed postoperative OH [13]. Furthermore, adult spinal deformity (ASD) surgery has been associated with persistent postoperative OH, defined as lasting ≥1 week, with an incidence of 9% [11,14].

Concerning risk factors for postoperative OH, poor preoperative AIS grades (A, B, or C) are particularly significant and independent predictors. Patients with AIS grades A, B, or C were markedly more likely to develop OH compared to those with grades D or E (43.5% vs. 7.2%). An AIS grade of A, B, or C increased the likelihood of postoperative OH by approximately sixfold [13]. In addition, traumatic injury mechanisms and posterior surgical approaches were also found to significantly influence the occurrence of OH in univariate analyses [13].

Neurodegenerative diseases, including Parkinson’s disease and multiple system atrophy, serve as independent predictors of postoperative OH, increasing the risk by approximately fourfold [14]. Furthermore, aging, often due to associated autonomic dysfunction, is also a recognized risk factor for OH [14].

The pathophysiology of postoperative OH is multifactorial, especially in the context of SCI. There is a strong association between cervical SCI and OH, primarily due to disruption of sympathetic tone caused by injury to descending preganglionic neurons, which leads to various cardiovascular abnormalities [13]. The severity of OH correlates with the degree of neurological deficit following SCI. Higher-level injuries (such as lesions above T6) are particularly critical, as they can impair sympathetic innervation to major vessels, thereby compromising the vasoconstrictive response mediated by the baroreflex [13].

Beyond direct neurological disruption, other factors contribute to OH. For instance, decreased muscle pump function in the lower limbs, resulting from inactivity of these muscles, leads to insufficient venous return and exacerbates OH [2,13,14]. Moreover, a reduction in blood volume due to dehydration or bleeding is a known mechanism that can worsen OH after SCI [2,14]. Surgical complications may also be implicated. Posterior pseudomeningocele can induce OH by decreasing intracranial pressure in the upright position, which triggers a compensatory drop in the mean arterial pressure to maintain cerebral perfusion, particularly in patients with impaired sympathetic tone due to SCI [15]. Anterior pseudomeningocele can cause OH by mechanically compressing the carotid body, leading to increased vagal tone, bradycardia, and syncope [16]. Cervical decompression surgery itself may contribute to autonomic instability. Decompression of the cervical cord can alter sympathetic pathways and disrupt descending autonomic regulation, thereby predisposing patients to postoperative OH. This pathophysiologic interplay highlights the importance of monitoring autonomic function following cervical laminoplasty, particularly in elderly patients with pre-existing neurological deficits.

Nonpharmacological interventions, particularly rehabilitation-based approaches, are generally recommended as the first-line treatment for OH before considering pharmacological therapy, and their effectiveness has been documented in various studies [10,17]. For instance, meta-analyses have indicated that lower limb compression can reduce postural SBP drops by approximately 9.83 mmHg; however, the quality of evidence supporting this intervention in elderly populations remains very low [10]. Furthermore, research focusing solely on SCI patients has yielded contradictory results regarding the efficacy of pressure devices such as abdominal binders and stockings [17]. This variability and the paucity of reliable evidence help to explain why such nonpharmacological measures often prove ineffective, as observed in our patient with complex post-surgical autonomic dysfunction. Despite these interventions, OH in our patient remained refractory, underscoring the limitations of conventional nonpharmacological strategies.

Functional electrical stimulation (FES) of the lower limbs has recently emerged as a promising modality, as it can enhance venous return by activating the muscle pump and potentially stabilize blood pressure during orthostatic stress. Although current evidence is preliminary, FES and other innovative rehabilitation techniques may represent future directions for managing refractory OH in patients with cervical SCI.

The lack of improvement in our patient’s OH is likely attributable to multiple interacting factors. Cervical SCI may have disrupted descending sympathetic pathways, impairing vascular tone and baroreflex-mediated regulation. Age-related decline in autonomic function and baroreceptor sensitivity further compounded this instability. Prolonged bed rest reduced lower limb muscle pump activity, limiting venous return. In addition, anemia and potential medication effects may have contributed to reduced cardiovascular resilience. Taken together, these multifactorial influences explain why OH persisted despite conventional nonpharmacological interventions.

Limitations

This report has several limitations. First, it is a single-case report, and therefore, causal inference and generalizability are limited. Second, standardized autonomic function tests (such as the Valsalva maneuver, deep breathing test, and catecholamine measurements) were not performed pre- and postoperatively, which made it impossible to precisely identify the lesion site or mechanism. Third, it was difficult to completely exclude confounding factors such as perioperative stress and dehydration, which may have contributed to the persistence of OH. Fourth, long-term follow-up after discharge was not available, and thus, whether OH ultimately improved remains unknown. Fifth, pharmacological agents such as midodrine, fludrocortisone, and droxidopa were not trialed, as the attending physician was concerned that these medications might cause excessive or unpredictable elevations in blood pressure, posing additional risks. Therefore, management was limited to nonpharmacological interventions.

Sixth, the rehabilitation intervention protocols (tilt angle, frequency, exercise intensity, etc.) were not sufficiently quantified, limiting reproducibility. Additionally, in this case, symptom severity and functional outcomes were evaluated using the Functional Independence Measure (FIM), but standardized quality of life (QoL) questionnaires such as the Orthostatic Hypotension Questionnaire (OHQ) or the Short Form Health Survey (36-Item) (SF-36) were not employed, and thus, the patient’s subjective burden and quality of life were not fully captured. These limitations highlight the need for future studies incorporating standardized autonomic testing, clearly defined rehabilitation protocols, validated QoL assessments, and long-term follow-up to better elucidate the underlying mechanisms and to establish evidence-based strategies for the management of postoperative OH.

## Conclusions

This case highlights the occurrence of refractory postoperative OH in an elderly patient with severe cervical compressive myelopathy (AIS grade C) after cervical laminoplasty, which persisted despite comprehensive nonpharmacological rehabilitation strategies. As a single-case report, the findings should not be overgeneralized; however, this presentation underscores the clinical significance of refractory OH in elderly patients with cervical SCI, where extensive spinal cord injury-induced sympathetic dysfunction, impaired muscle pump function, and age-related autonomic decline converge. Recognition of this multifactorial etiology is important for clinicians, emphasizing the need for careful monitoring and individualized management. Future studies incorporating standardized autonomic function tests, clearly defined rehabilitation protocols, and novel strategies such as FES are warranted to better elucidate underlying mechanisms and to establish evidence-based approaches for improving outcomes and quality of life in this vulnerable population.
